# Water Dynamics in Starch Based Confectionery Products including Different Types of Sugar

**DOI:** 10.3390/molecules27072216

**Published:** 2022-03-29

**Authors:** Esmanur İlhan, Pelin Poçan, Danuta Kruk, Miłosz Wojciechowski, Maciej Osuch, Roksana Markiewicz, Stefan Jurga, Mecit Halil Oztop

**Affiliations:** 1 Department of Food Engineering, Middle East Technical University, Ankara 06800, Turkey; esmanur@metu.edu.tr (E.İ.); mecit@metu.edu.tr (M.H.O.); 2 Department of Food Engineering, Faculty of Engineering and Architecture, Konya Food and Agriculture University, Konya 42080, Turkey; pelin.pocan@gidatarim.edu.tr; 3 Department of Physics & Biophysics, Faculty of Food Sciences, University of Warmia and Mazury in Olsztyn, Michala Oczapowskiego 4, 10-719 Olsztyn, Poland; maciej.osuch@uwm.edu.pl; 4 Faculty of Mathematics and Computer Science, University of Warmia and Mazury in Olsztyn, Sloneczna 54, 10-710 Olsztyn, Poland; wojciechowski@matman.uwm.edu.pl; 5 NanoBioMedical Centre, Adam Mickiewicz University, Wszechnicy Piastowskiej 3, 61-614 Poznan, Poland; roksana.markiewicz@amu.edu.pl (R.M.); stjurga@amu.edu.pl (S.J.)

**Keywords:** fast field cycling (FFC) NMR relaxometry, confectionery, starch, soy protein isolate, d-allulose, relaxation, dynamics

## Abstract

Starch-based confectionery products were prepared using different types of sugar. In addition to using different sugar, starch was replaced with soy protein isolate (SPI) in some of the products. ^1^H NMR spin-lattice relaxation experiments were performed for the collection of products in a broad frequency range from 4 KHz to 30 MHz to get insight into the influence of different sugar types and SPI on the dynamics of water in composite gel systems. The relaxation data have been decomposed into relaxation contributions associated with two different pools of water molecules characterized by different mobility. The translation dynamics of water molecules has been quantitatively described in terms of a dedicated relaxation model. The influence of the sample composition (the type of sugar and/or the presence of SPI) on the water mobility was thoroughly discussed. The results indicate that the addition of soy protein does not affect water dynamics for samples including sucrose. In addition, as the complementary measurements, physical properties of the products, such as the moisture content, water activity and texture, were investigated in terms of X-ray diffraction and thermogravimetric analysis.

## 1. Introduction

Confectionery gels are composed of high amounts of sugar components such as sucrose and glucose syrup, gelling agents such as starch, gelatin, or pectin along with food flavorings and colorings. In non-confectionery gel systems, gelation involves the dissolution of biopolymers in an aqueous environment and subsequent gelation via a crosslinking agent [1,2]. However, the addition of sugar greatly influences the standard gelation mechanism due to the low mobility of water and high solid fraction [3].

The high sucrose and glucose syrup content of confectionary gels is an increasing concern due to high sugar intake. Therefore, reducing sugar consumption leads one to use low and non-calorie sweeteners as a substitute for sucrose. Recently, it has been shown that the use of rare sugar, d-Allulose (a type of monosaccharide found in nature in small amounts), has yielded desirable characteristics in terms of processing and rheological properties of the confectionary products [4,5,6] with positive health effects [7,8,9,10]. d-allulose (C-3 epimer of fructose with a ketone group) has 70% of the sweetness of sucrose with a lower caloric value of 0.39 kcal/g due to poor digestion [11,12]. It has been shown that d-allulose exhibits different water-binding properties compared to other monosaccharides [13], and physical properties of the confectionery products can be affected by the water binding ability of sugars used in formulations. d-allulose has a wide array of biomedical applications, such as improving insulin resistance, its anti-obesity effects, its anti-inflammatory nature, and in regulating glucolipid metabolism [14,15]. It is also worth mentioning that d-allulose intake improves cholesterol metabolism, leading thus to a reduced risk of atherosclerotic plaque formation, which is considered a major cause of ischemic heart disease [16].

Proteins and polysaccharides are widely used as components of gel matrices. Depending on relative ratios of these components and the concentration of the exceeding polymer, one can observe segregation or complexation effects [17,18,19]. Therefore, a proper combination and concentration of polymers is essential for the formation of gel matrices and their stability. This subject becomes even more complex for confectionery gel systems containing several gelling agents, including proteins and polysaccharides [5,20,21,22,23,24,25].

In this work, we focused on the influence of d-allulose and soy protein isolate on the dynamic properties of the starch-based composite gel matrices and the relationship between the dynamical (molecular) and macroscopic features was exploited by nuclear magnetic resonance (NMR) relaxometry.

There is a growing interest in food science in applying NMR relaxometry as a tool enabling the linking of macroscopic properties of food products with dynamics on the molecular level. NMR relaxometry has been applied to enquire into the dynamical properties of several kinds of food, including eggs [26], where differences in water dynamics in different kinds of eggs and the influence of storage on dynamical properties of water were investigated. Whey-protein-based composite hydrogels [27], with different formulations, were examined to understand the dynamics of water molecules enclosed in the systems. One should also mention the works devoted to the aging of banana and spoilage of milk [28], the determination of the dynamics of virgin rape oil molecules [29], the characterization of balsamic vinegars of different aging processes [30], and the establishment of a relationship between translational diffusion coefficients and the viscosity of different kinds of oil for controlling the authenticity of oil products [31]. Furthermore, as far as sugar-containing products are considered, NMR relaxometry has recently been applied to investigate molecular properties of gelatin-based soft candies [32] in which the macroscopic properties of gelatin-based confections were correlated with water performance and provided methodological guidance on how to use the FFC NMR relaxometry to obtain a quantitative characterization of these products.

In the present work, the influence of d-allulose (replacing sucrose) and soy protein isolate on water mobility in starch-based confectionery products has been investigated by means of FFC NMR relaxometry. In addition, as a complementary method to FFC NMR relaxometry, thermal gravimetric analysis experiments (TGA), water activity, moisture content, and X-ray diffraction (XRD) analysis were also conducted, and the results were compared accordingly.

## 2. Results

### 2.1. NMR Relaxometry

**Figure 1:**
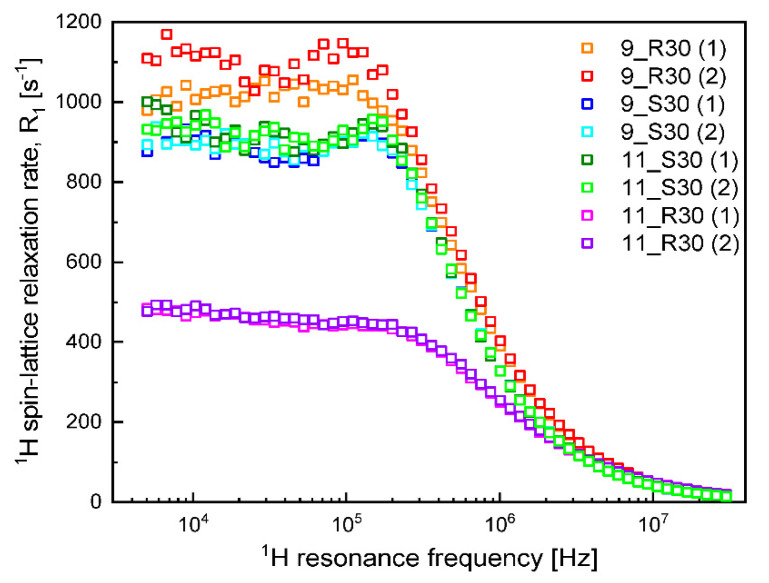
^1^ H spin-lattice relaxation data for the prepared samples.

The ^1^H spin-lattice relaxation data obtained for the set of samples listed in Table 1 are presented in Figure 1. Two observations can be made at this stage—the first one is that the data for the two samples of each kind ((1) and (2)) are in a good agreement. Moreover, the other replicates were also consistent with each other. The second observation is that the relaxation rates for 9S30 and 11S30 are very close, while the relaxation rates for 9_R30 and 11_R30 differ from them and are considerably different between each other.

^1^H relaxation processes are caused by magnetic dipole-dipole interactions. The interactions fluctuate in time as a result of molecular motion. As the relaxation experiments have been carried out in the frequency range encompassing five decades, the relaxation rates are associated with dynamical processes occurring on considerably different time scales—at low frequencies, one observes slow dynamics, while with increasing frequency a progressively faster dynamics is probed. In the simplest case of a single dynamical process contributing to the relaxation, the relaxation rate, $R_{1}\left(\omega \right)$ (ω) denotes the resonance frequency in angular frequency units), can be expressed as [27];

(1) $$R_{1}\left(\omega \right)=C_{DD}\left[\frac{\tau_{c}}{1+{\left(\omega \tau_{c}\right)}^{2}}+\frac{4\tau_{c}}{1+{\left(2\omega \tau_{c}\right)}^{2}}\right]\;$$

where $\tau_{c}$ denotes a characteristic time constant of the dynamical process, referred to as a correlation time, while $C_{DD}$ is the corresponding dipolar relaxation constant. Anticipating the results, it has turned out that the relaxation data of Figure 1 can be reproduced in terms of two dynamical processes and a frequency independent relaxation contribution:

(2) $$R_{1}\left(\omega \right)=C_{DD}^{s}\left[\frac{\tau_{c}^{s}}{1+{\left(\omega \tau_{c}^{s}\right)}^{2}}+\frac{4\tau_{c}^{s}}{1+{\left(2\omega \tau_{c}^{s}\right)}^{2}}\right]+C_{DD}^{f}\left[\frac{\tau_{c}^{f}}{1+{\left(\omega \tau_{c}^{f}\right)}^{2}}+\frac{4\tau_{c}^{f}}{1+{\left(2\omega \tau_{c}^{f}\right)}^{2}}\right]+A\;$$

The pairs of parameters $\tau_{c}^{s}$, $C_{DD}^{s}$ and $\tau_{c}^{f}$**,** $C_{DD}^{f}$ denote the correlation time and the corresponding dipolar relaxation constant for the slower and the faster dynamical processes, respectively. The frequency independent term, A, represents a relaxation contribution associated, in fact, with a motion that is so fast that its correlation time, $\tau_{c}$, fulfills the condition: $\omega \tau_{c}$ ≪ 1 and, consequently, the corresponding expression does not depend on frequency (in the frequency range exploited in this work).

**Figure 2:**
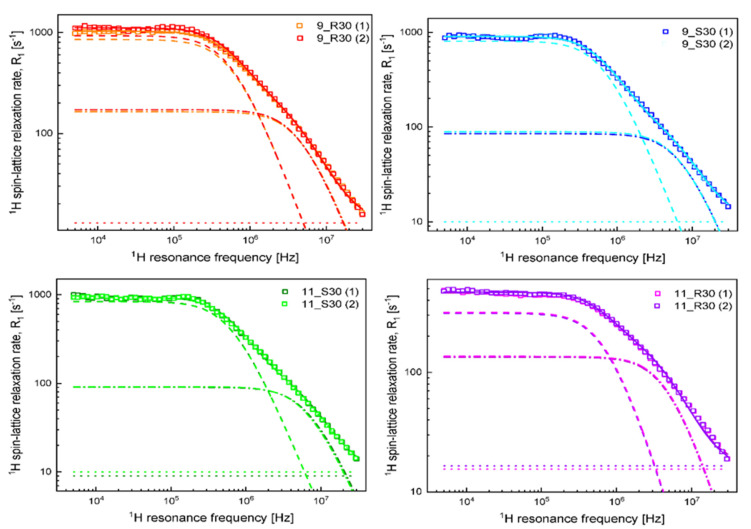
^1^H spin-lattice relaxation data for samples listed in Table 3. Solid lines—fits in terms of Equation (2) decomposed into the relaxation contributions associated with the slow dynamics (dashed lines), fast dynamics (dashed-dotted lines), and the frequency independent term (dotted lines).

The results of the analysis of the data in terms of Equation (2) are shown in Figure 2; the theoretical curves have been decomposed into the individual relaxation contributions.

The obtained parameters are collected in Table 1.

### 2.2. Water Activity, Moisture Content, Hardness, Thermogravimetric Analysis, and X-ray Diffraction

In addition to the NMR relaxation studies, water activity, moisture content and hardness for the different gel formulations have been measured. The results are collected in Table 2. The moisture content is described by the percentage of the ratio of the weight of water to the total weight of the material. The hardness values (N) represent peak forces during compression of the samples, while the temperatures at which the derivative off the mass loss show peaks (minima) are referred to as peak temperatures (°C).

**Figure 3:**
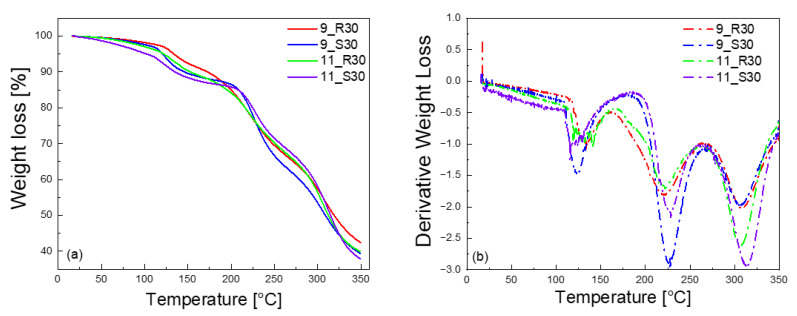
TGA results for 9_R30, 9_S30, 11_R30, and 11_S30 (**a**); derivatives of the mass loss versus temperature (**b**).

The measurements have been complemented by thermogravimetric analysis (TGA). The results are shown in Figure 3a,b, while the peak temperatures are included in Table 2.

**Figure 4:**
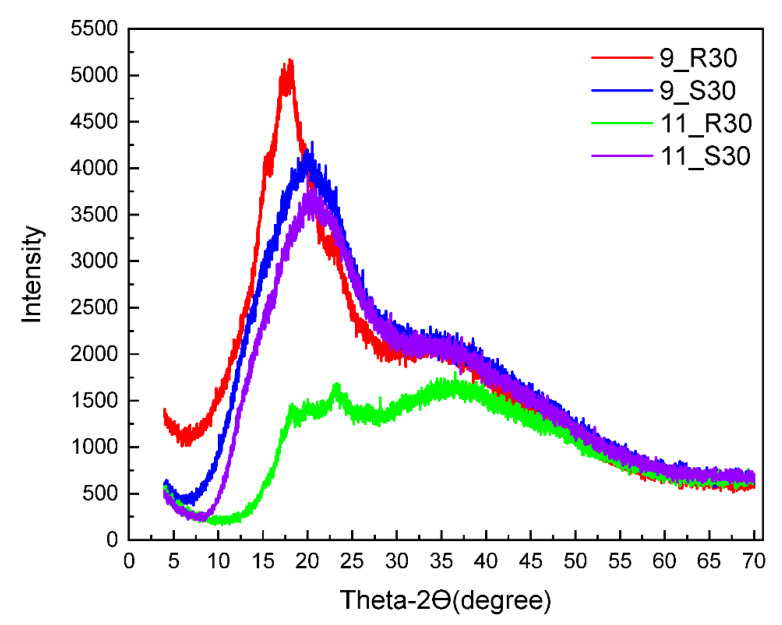
XRD patterns for 9_R30, 9_S30, 11_R30 and 11_S30.

To complete the characterization of the samples, X-ray diffraction (XRD) experiments have been performed. The XRD patterns are shown in Figure 4.

## 3. Discussion

One can clearly see from Figure 1 that at low frequencies, the ^1^H spin-lattice relaxation rates for 9_R30 are higher than for 9_S30—then with increasing frequency, the relaxation rates tend to coincide (in the high frequency range). The relaxation data have been interpreted in terms of a model assuming the presence of two (at least) pools of water molecules characterized by different mobility. The dynamics of both fractions is considerably slowed down by the confinement (interactions with the sugar fraction), nevertheless, to distinguish between them, we use the terminology “slow” and “fast”. The characteristic correlation time $\tau_{c}^{s}$ for the “slow” fraction of water molecules yields = 1.65 × 10^−7^ s for 9_R30, while the corresponding dipolar relaxation constant $C_{DD}^{s}$ yields 1.08 × 10^9^ Hz^2^. Analogous values for 9_S30 are given as $\tau_{c}^{s}$ = 1.44 × 10^−7^ s and $C_{DD}^{s}$ = 1.13 × 10^9^ Hz^2^. This comparison shows that the parameters characterizing the “slow” fraction of water in 9_R30 and 9_S30 are similar. The correlation times for the “fast” water fraction is equal to $\tau_{c}^{f}$ = 2.03 × 10^−7^ s for 9_R30 and $\tau_{c}^{f}$ = 1.35 × 10^−7^ s for 9_S30. This implies that dynamics of the “fast” water fraction for 9_R30 is slower than for 9_S30. It is worth noting at this stage that the dynamics of the “slow” fraction is in a good approximation slower by an order of magnitude compared to the dynamics of the “fast” water fraction. The dipolar relaxation constant $C_{DD}^{f}$ for 9_R30 is 1.67 × 10^9^ Hz^2^, while for 9_S30 it yields 1.30 × 10^9^ Hz^2^. Dipolar relaxation constants include (are proportional to) the factor Pq, where P is the mole faction of water in the bound position, while q denotes the coordination number [26]. This implies that the product Pq for the “fast” water fraction is larger for 9_R30 than for 9_S30.

Following this line, the relaxation data (and, consequently, the parameters) for 11_S30 are very similar to those for 9S_30: for the “slow” water fraction in 11_S30, it has been obtained: $\tau_{c}^{s}$ = 1.50 × 10^−7^ s, $C_{DD}^{s}$ = 1.12 × 10^9^ Hz^2^, while for the “fast” water fraction, the parameters yield: $\tau_{c}^{f}$ = 1.36 × 10^−8^ s, $C_{DD}^{f}$ = 1.34 × 10^9^ Hz^2^. The results show that the addition of soy protein (2%) does not affect the water dynamics as long as the samples include sucrose. When the sucrose is replaced by d-allulose in the soy protein containing samples, the relaxation rates become much smaller (Figure 1), and the corresponding parameters show significant differences. Comparing the parameters for 11_S30 with those for 11_R30, one sees that while the correlation time $\tau_{c}^{s}$ = 1.32 × 10^−7^ s for 11_R30 for the “slow” water fraction is close to the corresponding value for 11_S30, the dipolar relaxation constant for 11_R30, $C_{DD}^{s}$ = 4.78 × 10^8^ Hz^2^, is lower by more than la factor of two compared to the corresponding value for 11_S30. As far as the “fast” water fraction is concerned, the dipolar relaxation constant for 11_R30, $C_{DD}^{f}$ = 1.44 × 10^9^ Hz^2^ is close to the value for 11S-30, while the correlation time $\tau_{c}^{f}$ = 1.87 × 10^−8^ s for 11R_30 is somewhat longer than for 11S_30. As the dipolar relaxation constant for the “slow” water fraction in 11_R30 is also by more than a factor of two lower than the corresponding value for 9R_30, one can conclude that the mole fraction of bound water molecules for 11_R30 is by more than a factor of two lower than for 9_R30 (both samples include d-allulose, so the coordination number, q, is the same for both cases) and the difference is solely caused by the presence of soy protein in the small amount of 2%. One should also comment about the frequency independent term, C. This relaxation contribution represents a dynamical process that occurs on a fast time scale—of the order of a couple of ns or faster. Consequently, as the condition: $\omega \tau_{c}\ll 1$ (where $\tau_{c}$ denotes the corresponding correlation time) is fulfilled, in that case contribution becomes frequency independent. This relaxation term likely originates from several processes: dynamics of a “free” water fraction (a fraction of water molecules, the dynamics of which is not so significantly affected by interactions with the sugar and protein molecules) and/or dynamics of functional groups of sugar molecules. We would prefer not to speculate on this subject, but it is worthy of note that the A term is higher in the presence of d-allulose.

The d-allulose containing samples (9_R30 and 11_R30) are characterized by lower water activity and moisture content compared to their sucrose containing counterparts. In [5], it has been stated that the water-binding ability of d-allulose is lower than that of sucrose. Consequently, it has been argued that during the preparation of the sugar syrup mixture, d-allulose-containing formulations might have lost more water due to evaporation, which could have resulted in lower “free” water and moisture content in the final product [5]. Independently of these considerations, the water activity and moisture content vary in rather narrow ranges that clearly indicates that these quantities are not affected by the population of the “slow” water fraction.

One should point out the relationship between the relaxation properties and the XRD patterns for the four formulations. XRD experiments were performed to understand the crystallinity of the confectionary formulations. The highest and the most narrow XRD peak has been observed for 9_R30—this corresponds to the fastest relaxation process (fast relaxation is characteristic of materials of high crystallinity). Then the XRD patterns for 9_S30 and 11_S30 almost overlap— and so do the relaxation data. Eventually the broadest XRD peak is observed for 11_R30 (indicating a distribution of crystals, i.e., polydispersity of the sample), corresponding to the slowest relaxation reflected by the relatively small population of the “slow” water fraction.

The presence of soy proteins increases the hardness independently of the sugar (d-allulose or sucrose)—this is clearly seen from Table 3 (6.07 for 9_R30 versus 2.58 for 11_R30 and 23.92 for 9_S30 versus 6.08 for 11_S30). At the same time, one observes that hardness decreases upon replacing sucrose by d-allulose (23.92 for 9_S30 versus 6.07 for 9_R30 and 6.08 for 11_S30 versus 2.58 for 11_R30) independently of the presence of soy proteins. Amine groups present in protein chains react with glucose and d-Allulose upon heat treatment through Maillard reaction. The addition of soy proteins to the formulation means introducing amine groups that react with the monosaccharides. The reaction results in the formation of covalent cross-links within the protein network [33] that might be the reason for the increase of hardness in the presence of soy proteins.

A closer inspection of the TGA curves and their derivatives indicates a three-step mass loss for all samples in the temperature range from 25 °C to 350 °C. The mass loss in the first (low temperature) stage can be attributed to losing water [34], while the high temperature ones can be associated with the decomposition of polymers and organic compounds in the formulations. The temperature position of the first minimum in the derivative curves e (starting from the low temperature) reveals that for sucrose-containing samples (11_S30 and 9_S30), water escapes from the system at 119 °C and 123 °C, respectively. On the other hand, for the d-allulose containing samples (11_R30 and 9_R30), water escapes from the system at 141 °C and 133 °C, which are slightly higher compared to the sucrose containing samples.

## 4. Materials and Methods

### 4.1. Sample Preparation

Four kinds of starch-based gels were prepared, and are shown in Table 3. The first pair (9_R30 and 9_S30) includes 9% starch and 2% soy protein—the difference is in replacing the sucrose (present in 9_S30) by d-allulose (9_R30). The second pair (11_R30 and 11_S30) includes 11% starch (no soy protein) and again glucose (11_S30) and d-allulose (11_R30). For each case, two samples were prepared. In all cases, the remaining contribution (29%) is water.

The gels were prepared according to the method described in [5]. The amount of water used before cooking was 29% of the total mass weight. The water amount was divided into two to gelatinize the starch and to prepare the sugar solutions. In the first part, starch was mixed with water in the proportion of 1:2 (starch: water) and gelatinized in an oil bath at 140 °C for 5 min. The sugar syrup—powder sugar mixture was mixed in a glass beaker with the remaining water and boiled up to 115 °C. After that, the gelatinized starch was mixed with the syrup mixture at 115 °C. The soy protein isolate was added at this stage (for the formulations including soy protein isolate) and homogenized at 10,000 rpm for 1 min (WiseTisHG-15D homogenizer, Wertheim, Germany). The cooking continued in an oil bath at 140 °C until the mixture attained 75°Brix value. oBrix values of the formulations were measured by a hand refractometer (Hanna, HI96801, Smithfield, RI, USA). The hot mixture was then poured into starch molds with dimensions of 2.5 × 2.5 × 2 cm and kept at 38 °C for 36 h.

### 4.2. Experimental Methods

The ^1^H spin-lattice relaxation experiments have been carried out in the frequency range from 5 kHz to 30 MHz using a STELAR (Mede, Italy) Spinmaster relaxometer. The experiments have been performed at 25 °C with a temperature accuracy of 1 °C. For each resonance frequency, 16 points have been collected for the magnetization curve (^1^H magnetization versus time). The relaxation process has turned out to be single-exponential; the corresponding magnetization curves are presented in Supplementary Material. Consequently, the 1H spin-lattice relaxation rates have been obtained from single-exponential fits of the magnetization versus time curves for each resonance frequency.

Thermogravimetric analysis (TGA) was performed with a Perkin Elmer Pyris1 (Perkin Elmer, MA, USA) in the temperature range from 25 °C to 350 °C with a heating rate of 5 °C/min, under nitrogen.

X-ray diffraction experiments were performed with a Rigaku Ultima-IV X-Ray Diffractometer (Japan). The data were collected by the method of [35] in the range of 4–70 °C.

Hardness of the starch-based gels was measured by using a Texture Analyzer (Lloyd Instruments, TA Plus, Hants, UK). A 35-mm cylinder shape probe of a diameter of 1 cm and load cell of 50 N was attached to the instrument. The samples were compressed twice with 100 mm/min pretest speed. For the data analysis, NEXIGEN texture analysis software was used.

Moisture content of the samples was measured at 70 °C for 4 h in a vacuum oven (DAIHAN, Wonju, Korea). Weight loss of the samples was recorded, and the moisture content of each sample was calculated on that basis.

An Aqualab 4 TE (METER Group, Pullman, WA, USA) was used to measure the water activities of the samples. The experiments were conducted at 25 °C.

## 5. Conclusions

^1^H spin-lattice relaxation data of starch-based, soy protein containing composite gel systems were collected in a wide frequency range (4 kHz to 30 MHz) and quantitatively analyzed. Two water fractions referred as the slow-water fraction and the fast-water fraction have been identified by finding the characteristic correlation time and the corresponding dipolar relaxation constant. The results show that the addition of soy protein (2%) does not affect the water dynamics as long as the samples include sucrose. When the sucrose is replaced by d-allulose in the soy protein containing samples, the relaxation rates become much smaller, and the corresponding parameters show significant differences. As the dipolar relaxation constant for the “slow” water fraction in d-allulose containing samples, the mole fraction of bound water molecules is lower by more than a factor of two for those where only starch is used as a gelling agent.

In this study, the unique potential of NMR relaxometry was exploited to get insight into water dynamics in the gel network. It should be pointed out that NMR relaxometry is a very useful method to investigate the properties of composite gels that are very common in food systems.

## Figures and Tables

**Table 1:** Parameters obtained from the analysis of the relaxation data in terms of Equation (2).

Sample	$C_{DD}^{s}\;[{\mathrm{Hz}}^{2}]$	$\tau_{c}^{s}\;[s]$	$C_{DD}^{f}[{\mathrm{Hz}}^{2}]$	$\tau_{c}^{f}\;[s]$	$A\;[s^{-1}]$
9_R30 (1)	1.06 × 10^9^ ± 1.02 × 10^7^	1.61 × 10^7^ ± 3.67 × 10^−8^	1.67 × 10^9^ ± 4.55 × 10^7^	1.97 × 10^−8^ ± 2.90 × 10^−9^	13.1
9_R30 (2)	1.10 × 10^9^ ± 1.43 × 10^7^	1.69 × 10^−7^ ± 5.18 × 10^−8^	1.67 × 10^9^ ± 6.23 × 10^7^	2.06 × 10^−8^ ± 4.23 × 10^−9^	13.1
9_S30 (1)	1.14 × 10^9^ ± 1.25 × 10^7^	1.43 × 10^−7^ ± 4.02 × 10^−8^	1.29 × 10^9^ ± 1.32 × 10^8^	1.32 × 10^−8^ ± 4.70 × 10^−9^	10.0
9_S30 (2)	1.12 × 10^9^ ± 1.01 × 10^7^	1.45 × 10^−7^ ± 3.33 × 10^−8^	1.30 × 10^9^ ± 9.72 × 10^7^	1.37 × 10^−8^ ± 3.81 × 10^−9^	10.0
11_R30 (1)	4.71 × 10^8^ ± 6.84 × 10^6^	1.32 × 10^−7^ ± 4.52 × 10^−8^	1.42 × 10^9^ ± 1.84 × 10^7^	1.88 × 10^−8^ ± 1.86 × 10^−9^	16.5
11_R30 (2)	4.84 × 10^8^ ± 6.91 × 10^6^	1.31 × 10^−7^ ± 4.45 × 10^−8^	1.46 × 10^9^ ± 1.79 × 10^7^	1.86 × 10^−8^ ± 1.84 × 10^−9^	15.5
11_S30 (1)	1.12 × 10^9^ ± 1.25 × 10^7^	1.50 × 10^−7^ ± 4.35 × 10^−8^	1.36 × 10^9^ ± 1.38 × 10^8^	1.33 × 10^−8^ ± 4.81 × 10^−9^	9.0
11_S30 (2)	1.11 × 10^9^ ± 1.13 × 10^7^	1.50 × 10^−7^ ± 3.89 × 10^−8^	1.31 × 10^9^ ± 1.10 × 10^8^	1.40 × 10^−8^ ± 4.36 × 10^−9^	10.1

**Table 2:** Water activity (a_w), moisture content (MC %), hardness (N) and peak temperatures (°C) for different formulations.

Sample Name	aw	MC %	Hardness (N)	Peak Temperature (°C)
**9_R30**	0.54 ± 0.00 ^d^	12.19 ± 0.00 ^c^	06.07 ± 0.57 ^b^	133.68 ± 0.71 ^ab^
**9_S30**	0.66 ± 0.00 ^b^	14.09 ± 0.01 ^b^	23.92 ± 0.81 ^a^	123.15 ± 3.08 ^bc^
**11_R30**	0.61 ± 0.01 ^c^	13.85 ± 0.07 ^b^	02.58 ± 0.24 ^c^	141.07 ± 0.49 ^a^
**11_S30**	0.69 ± 0.00 ^a^	14.94 ± 0.01 ^a^	06.08 ± 0.11 ^b^	119.75 ± 5.72 ^c^

Means within the same column, followed by the different small letters are significantly different for each sample (*p* < 0.05).

**Table 3:** Composition of the formulations.

Name	Starch (%)	D-Allulose (%)	Sucrose (%)	Corn Syrup (%)	Soy Protein (%)
9_R30	9	30	0	30	2
9_S30	9	0	30	30	2
11_R30	11	30	0	30	0
11_S30	11	0	30	30	0

## Data Availability

Not applicable.

## Supplementary Materials

The following supporting information can be downloaded at: https://www.mdpi.com/article/10.3390/molecules27072216/s1, Figures S1–S8: Normalized magnetization curves.

## Sample Availability

Samples of the compounds are available from the authors.
