# Supplementary material for: Water Dynamics in Starch Based Confectionery Products including Different Types of Sugar
Source: Molecules. 2022 Mar 29;27(7):2216. doi: 10.3390/molecules27072216 (PMC9000446; doi:10.3390/molecules27072216)
Supplement: Supplementary file 1 [file molecules-27-02216-s001.zip › molecules-1649432-supplementary.pdf]

Supplementary Material

FigureS1–S8: Normalized magnetization curves.

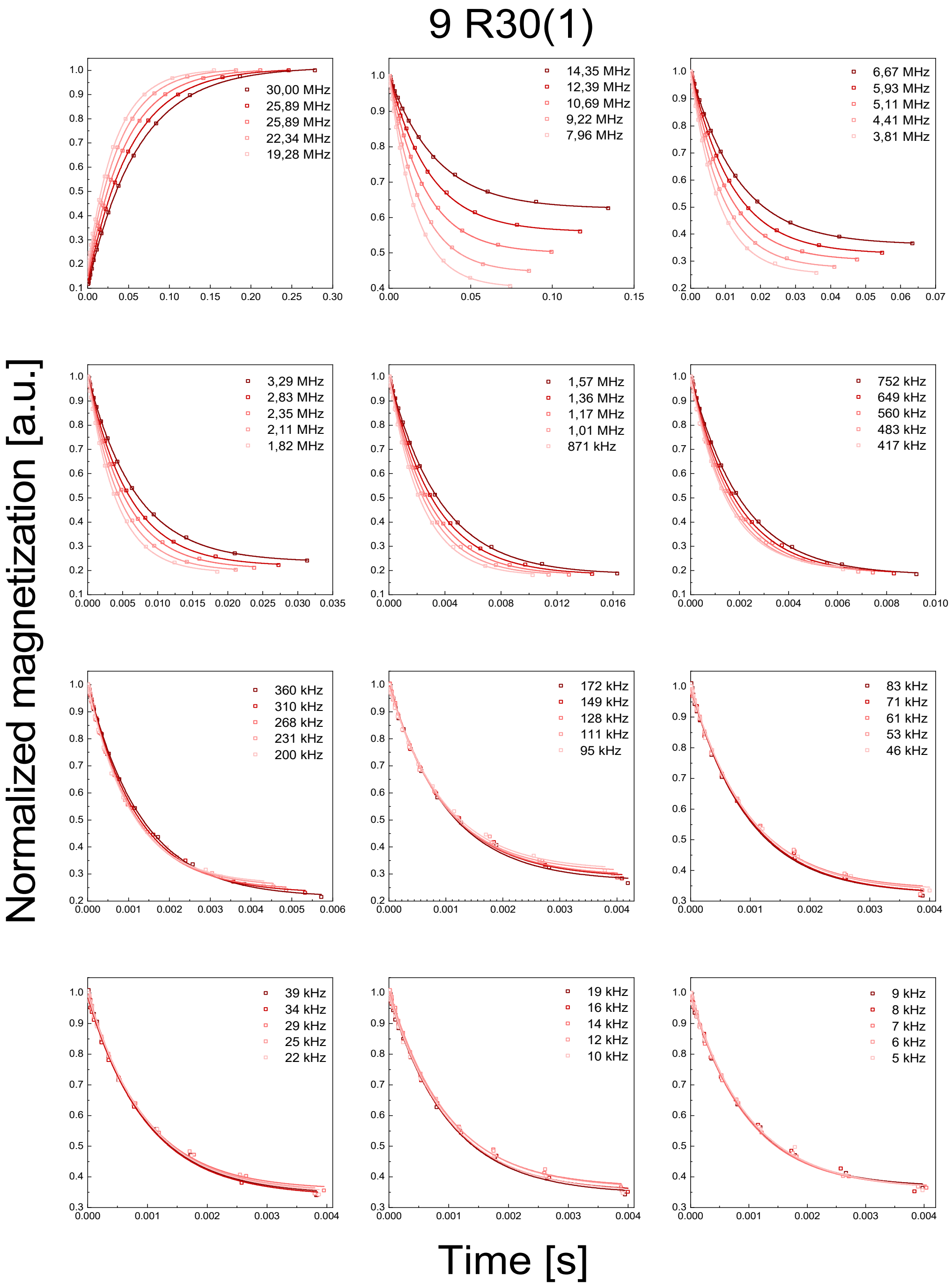

9 R30(2)

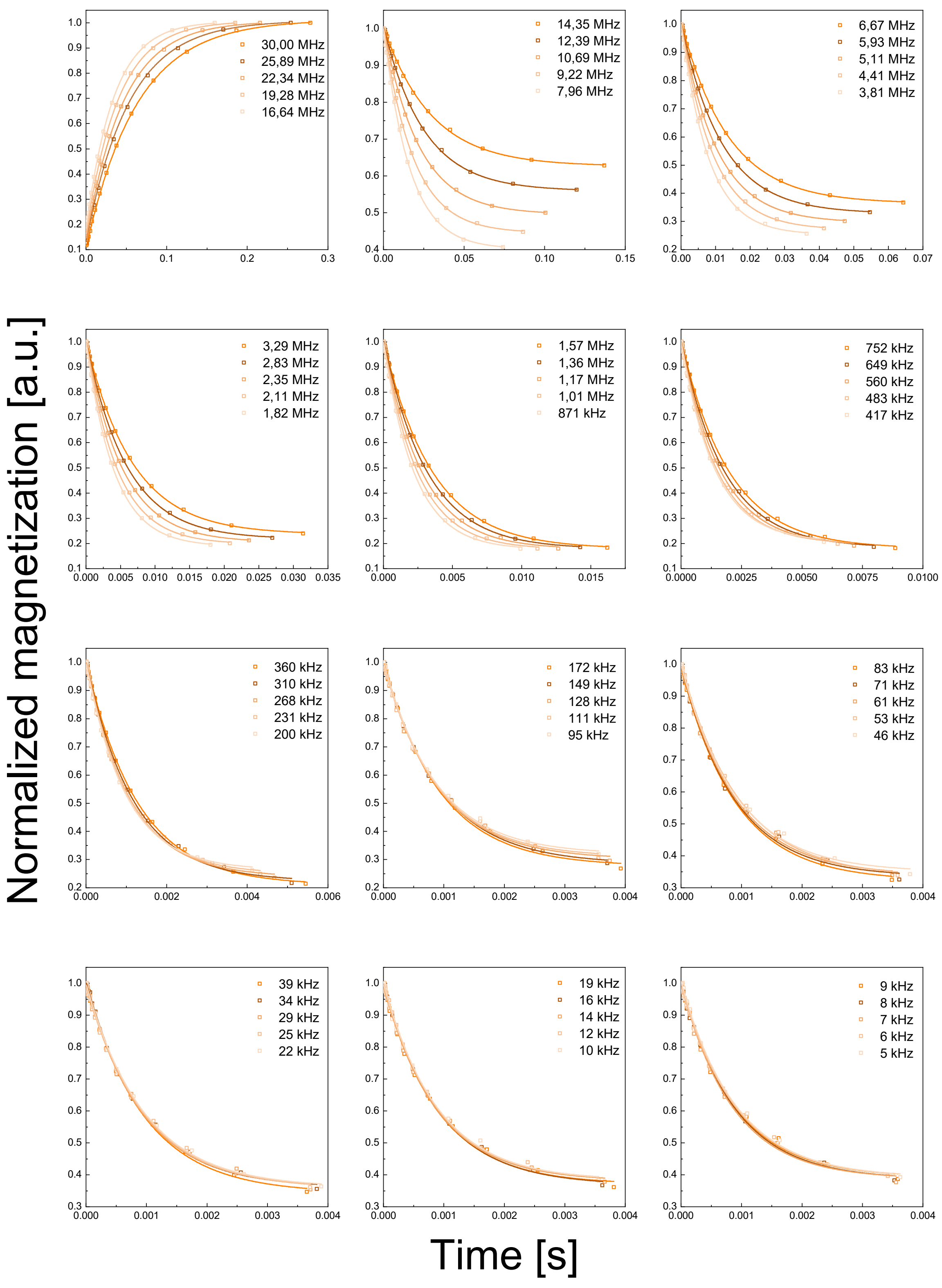
Normalized magnetization [a.u.]
Time [s]
30,00 MHz
25,89 MHz
22,34 MHz
19,28 MHz
16,64 MHz
14,35 MHz
12,39 MHz
10,69 MHz
9,22 MHz
7,96 MHz
6,67 MHz
5,93 MHz
5,11 MHz
4,41 MHz
3,81 MHz
3,29 MHz
2,83 MHz
2,35 MHz
2,11 MHz
1,82 MHz
1,57 MHz
1,36 MHz
1,17 MHz
1,01 MHz
871 kHz
752 kHz
649 kHz
560 kHz
483 kHz
417 kHz
360 kHz
310 kHz
268 kHz
231 kHz
200 kHz
172 kHz
149 kHz
128 kHz
111 kHz
95 kHz
83 kHz
71 kHz
61 kHz
53 kHz
46 kHz
39 kHz
34 kHz
29 kHz
25 kHz
22 kHz
19 kHz
16 kHz
14 kHz
12 kHz
10 kHz
9 kHz
8 kHz
7 kHz
6 kHz
5 kHz

9 S30(1)

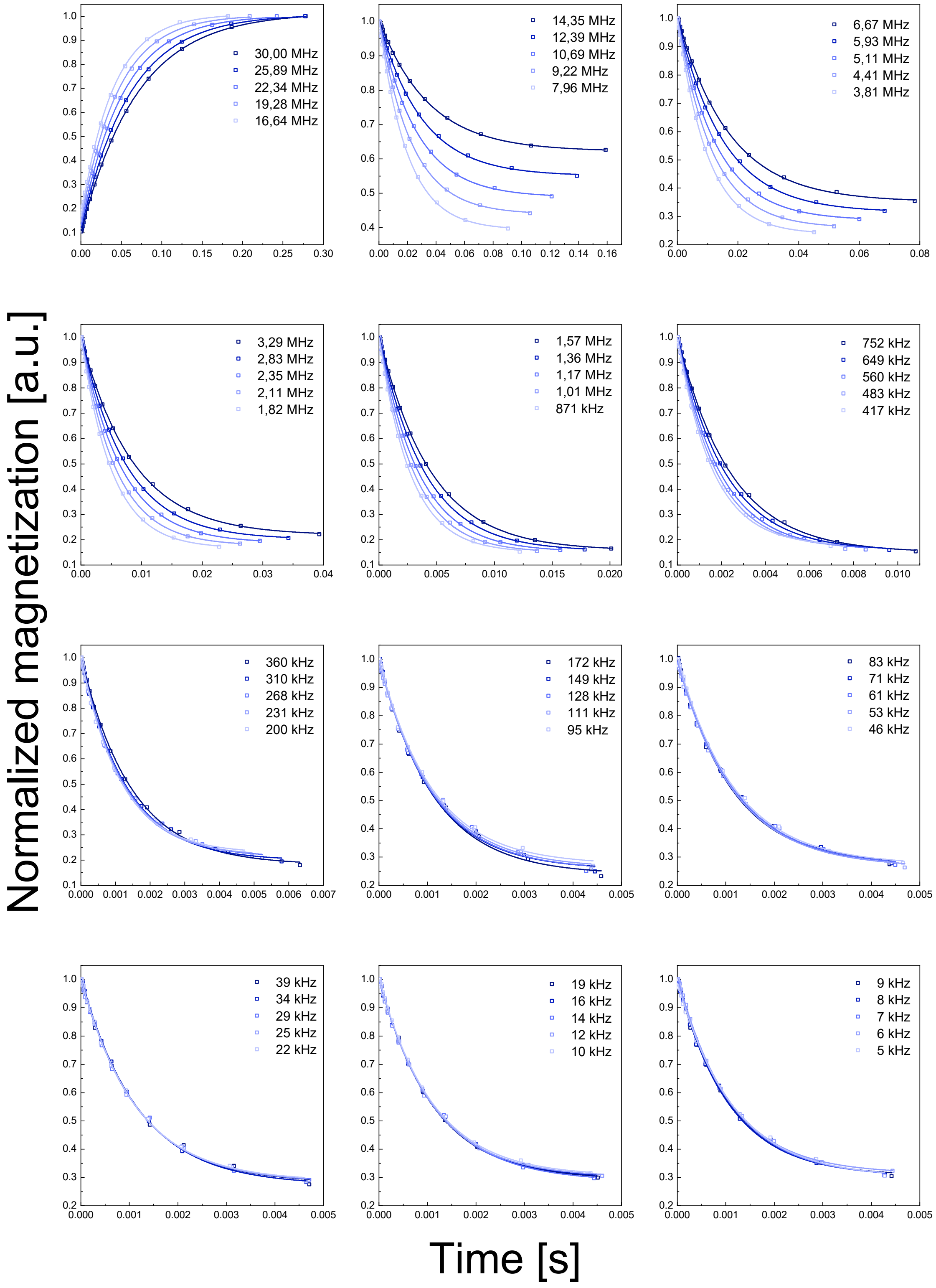
Normalized magnetization [a.u.]
Time [s]
30,00 MHz
25,89 MHz
22,34 MHz
19,28 MHz
16,64 MHz
14,35 MHz
12,39 MHz
10,69 MHz
9,22 MHz
7,96 MHz
6,67 MHz
5,93 MHz
5,11 MHz
4,41 MHz
3,81 MHz
3,29 MHz
2,83 MHz
2,35 MHz
2,11 MHz
1,82 MHz
1,57 MHz
1,36 MHz
1,17 MHz
1,01 MHz
871 kHz
752 kHz
649 kHz
560 kHz
483 kHz
417 kHz
360 kHz
310 kHz
268 kHz
231 kHz
200 kHz
172 kHz
149 kHz
128 kHz
111 kHz
95 kHz
83 kHz
71 kHz
61 kHz
53 kHz
46 kHz
39 kHz
34 kHz
29 kHz
25 kHz
22 kHz
19 kHz
16 kHz
14 kHz
12 kHz
10 kHz
9 kHz
8 kHz
7 kHz
6 kHz
5 kHz

9 S30(2)

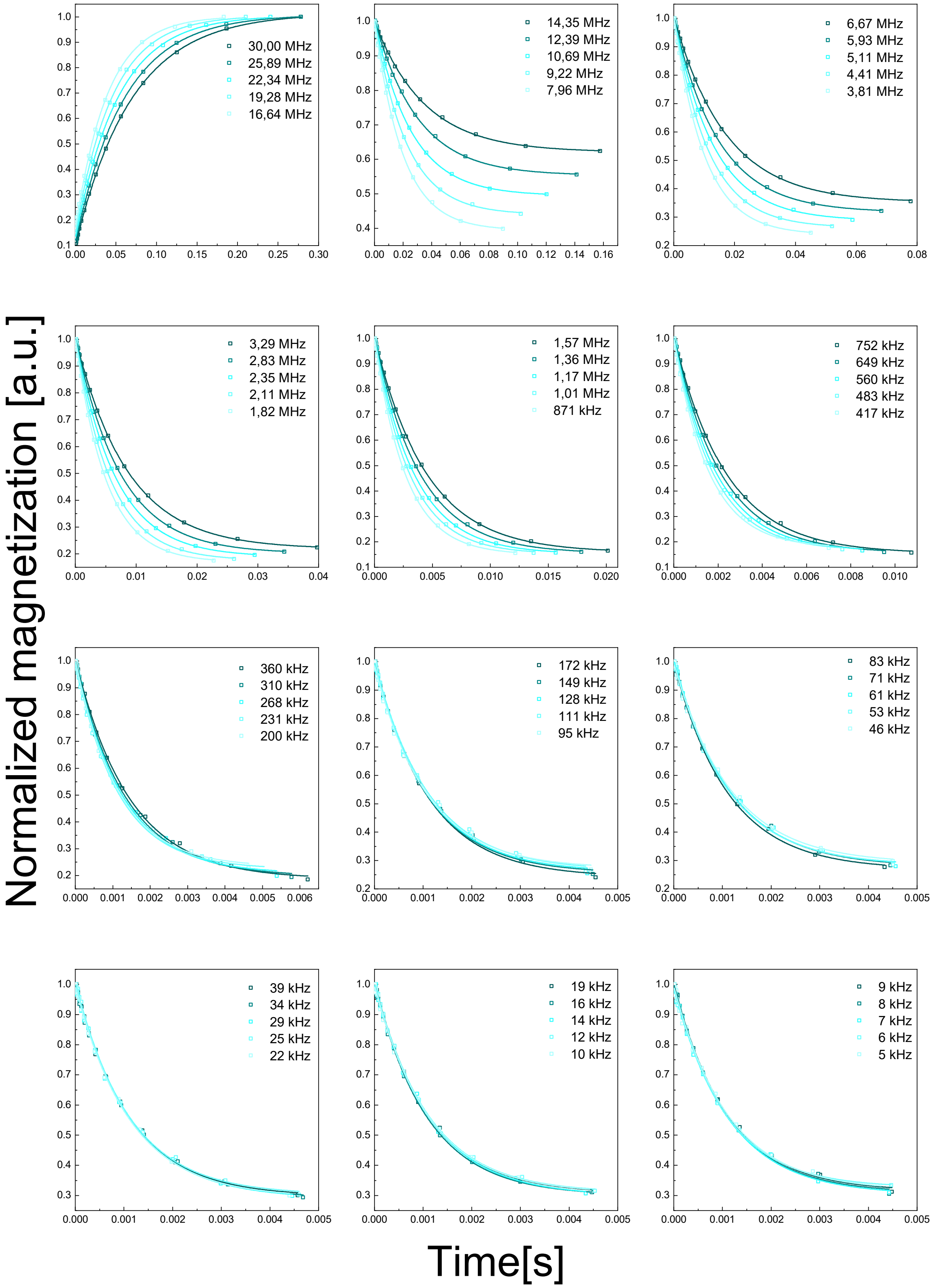

Normalized magnetization [a.u.]
Time[s]
30,00 MHz
25,89 MHz
22,34 MHz
19,28 MHz
16,64 MHz
14,35 MHz
12,39 MHz
10,69 MHz
9,22 MHz
7,96 MHz
6,67 MHz
5,93 MHz
5,11 MHz
4,41 MHz
3,81 MHz
3,29 MHz
2,83 MHz
2,35 MHz
2,11 MHz
1,82 MHz
1,57 MHz
1,36 MHz
1,17 MHz
1,01 MHz
871 kHz
752 kHz
649 kHz
560 kHz
483 kHz
417 kHz
360 kHz
310 kHz
268 kHz
231 kHz
200 kHz
172 kHz
149 kHz
128 kHz
111 kHz
95 kHz
83 kHz
71 kHz
61 kHz
53 kHz
46 kHz
39 kHz
34 kHz
29 kHz
25 kHz
22 kHz
19 kHz
16 kHz
14 kHz
12 kHz
10 kHz
9 kHz
8 kHz
7 kHz
6 kHz
5 kHz

11 S30(1)

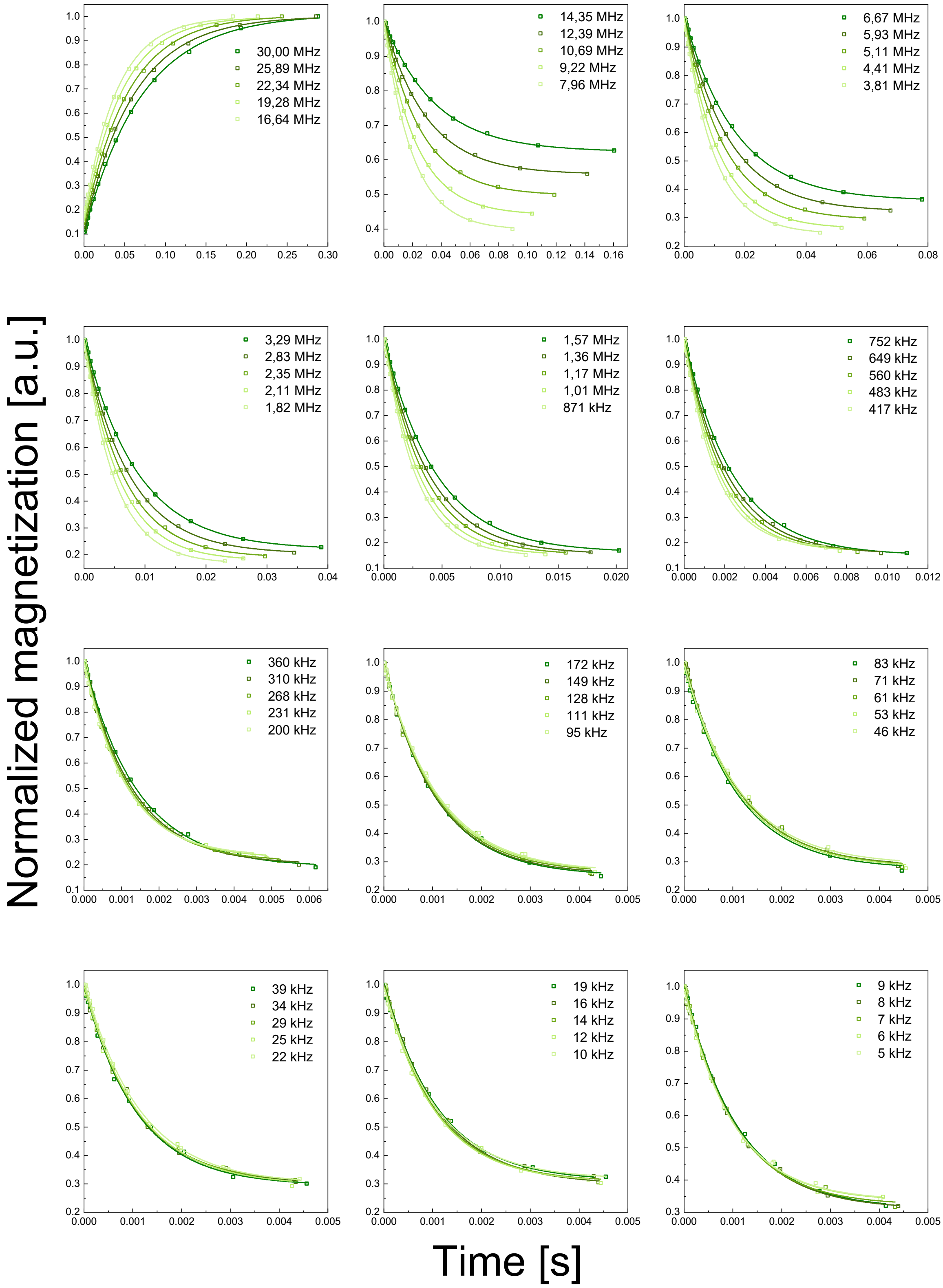
30,00 MHz
25,89 MHz
22,34 MHz
19,28 MHz
16,64 MHz
14,35 MHz
12,39 MHz
10,69 MHz
9,22 MHz
7,96 MHz
6,67 MHz
5,93 MHz
5,11 MHz
4,41 MHz
3,81 MHz
3,29 MHz
2,83 MHz
2,35 MHz
2,11 MHz
1,82 MHz
1,57 MHz
1,36 MHz
1,17 MHz
1,01 MHz
871 kHz
752 kHz
649 kHz
560 kHz
483 kHz
417 kHz
360 kHz
310 kHz
268 kHz
231 kHz
200 kHz
172 kHz
149 kHz
128 kHz
111 kHz
95 kHz
83 kHz
71 kHz
61 kHz
53 kHz
46 kHz
39 kHz
34 kHz
29 kHz
25 kHz
22 kHz
19 kHz
16 kHz
14 kHz
12 kHz
10 kHz
9 kHz
8 kHz
7 kHz
6 kHz
5 kHz
Normalized magnetization [a.u.]
Time [s]

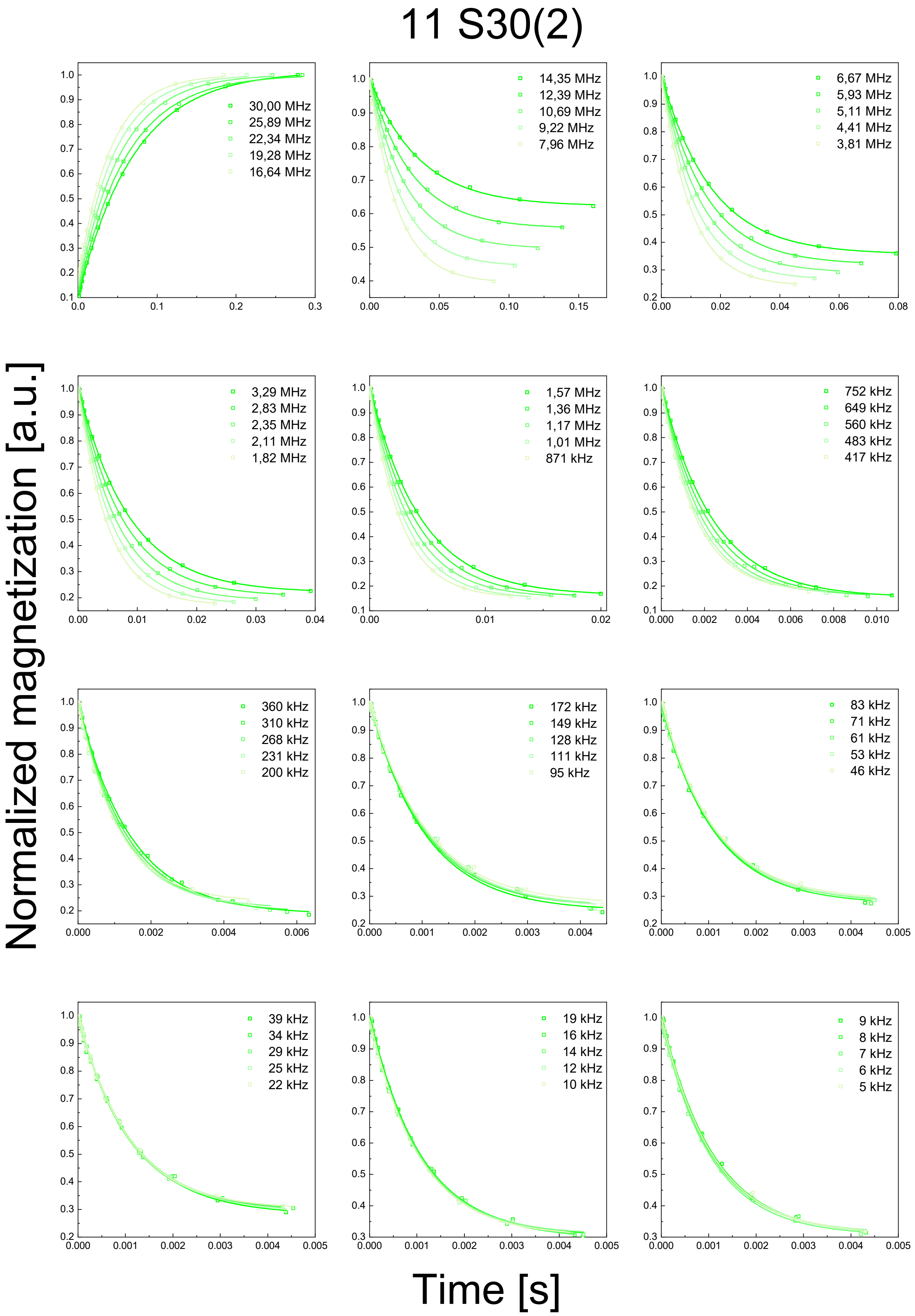

11 S30(2)
Normalized magnetization [a.u.]
Time [s]
30,00 MHz
25,89 MHz
22,34 MHz
19,28 MHz
16,64 MHz
14,35 MHz
12,39 MHz
10,69 MHz
9,22 MHz
7,96 MHz
6,67 MHz
5,93 MHz
5,11 MHz
4,41 MHz
3,81 MHz
3,29 MHz
2,83 MHz
2,35 MHz
2,11 MHz
1,82 MHz
1,57 MHz
1,36 MHz
1,17 MHz
1,01 MHz
871 kHz
752 kHz
649 kHz
560 kHz
483 kHz
417 kHz
360 kHz
310 kHz
268 kHz
231 kHz
200 kHz
172 kHz
149 kHz
128 kHz
111 kHz
95 kHz
83 kHz
71 kHz
61 kHz
53 kHz
46 kHz
39 kHz
34 kHz
29 kHz
25 kHz
22 kHz
19 kHz
16 kHz
14 kHz
12 kHz
10 kHz
9 kHz
8 kHz
7 kHz
6 kHz
5 kHz

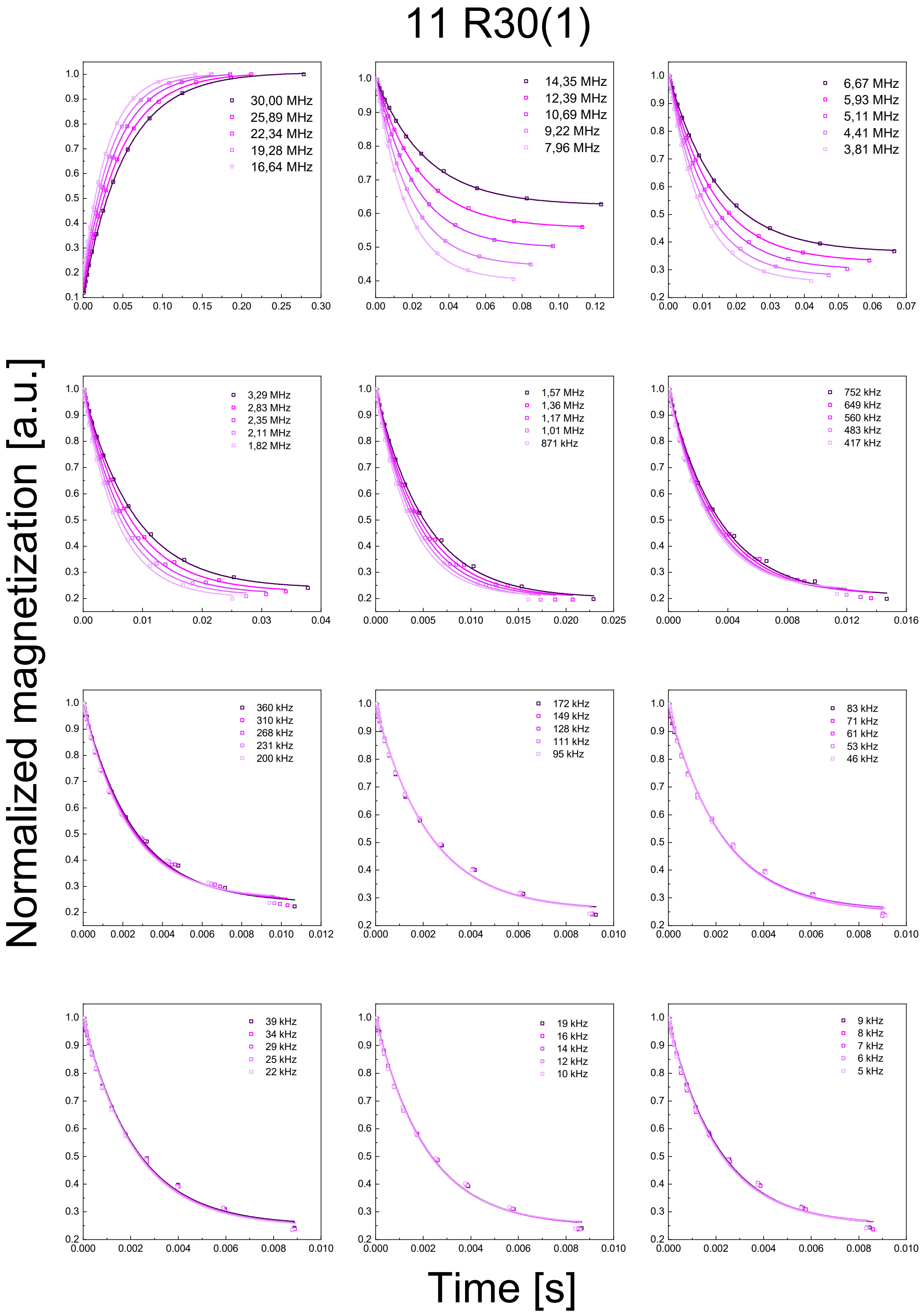

11 R30(1)
Normalized magnetization [a.u.]
Time [s]
30,00 MHz
25,89 MHz
22,34 MHz
19,28 MHz
16,64 MHz
14,35 MHz
12,39 MHz
10,69 MHz
9,22 MHz
7,96 MHz
6,67 MHz
5,93 MHz
5,11 MHz
4,41 MHz
3,81 MHz
3,29 MHz
2,83 MHz
2,35 MHz
2,11 MHz
1,82 MHz
1,57 MHz
1,36 MHz
1,17 MHz
1,01 MHz
871 kHz
752 kHz
649 kHz
560 kHz
483 kHz
417 kHz
360 kHz
310 kHz
268 kHz
231 kHz
200 kHz
172 kHz
149 kHz
128 kHz
111 kHz
95 kHz
83 kHz
71 kHz
61 kHz
53 kHz
46 kHz
39 kHz
34 kHz
29 kHz
25 kHz
22 kHz
19 kHz
16 kHz
14 kHz
12 kHz
10 kHz
9 kHz
8 kHz
7 kHz
6 kHz
5 kHz

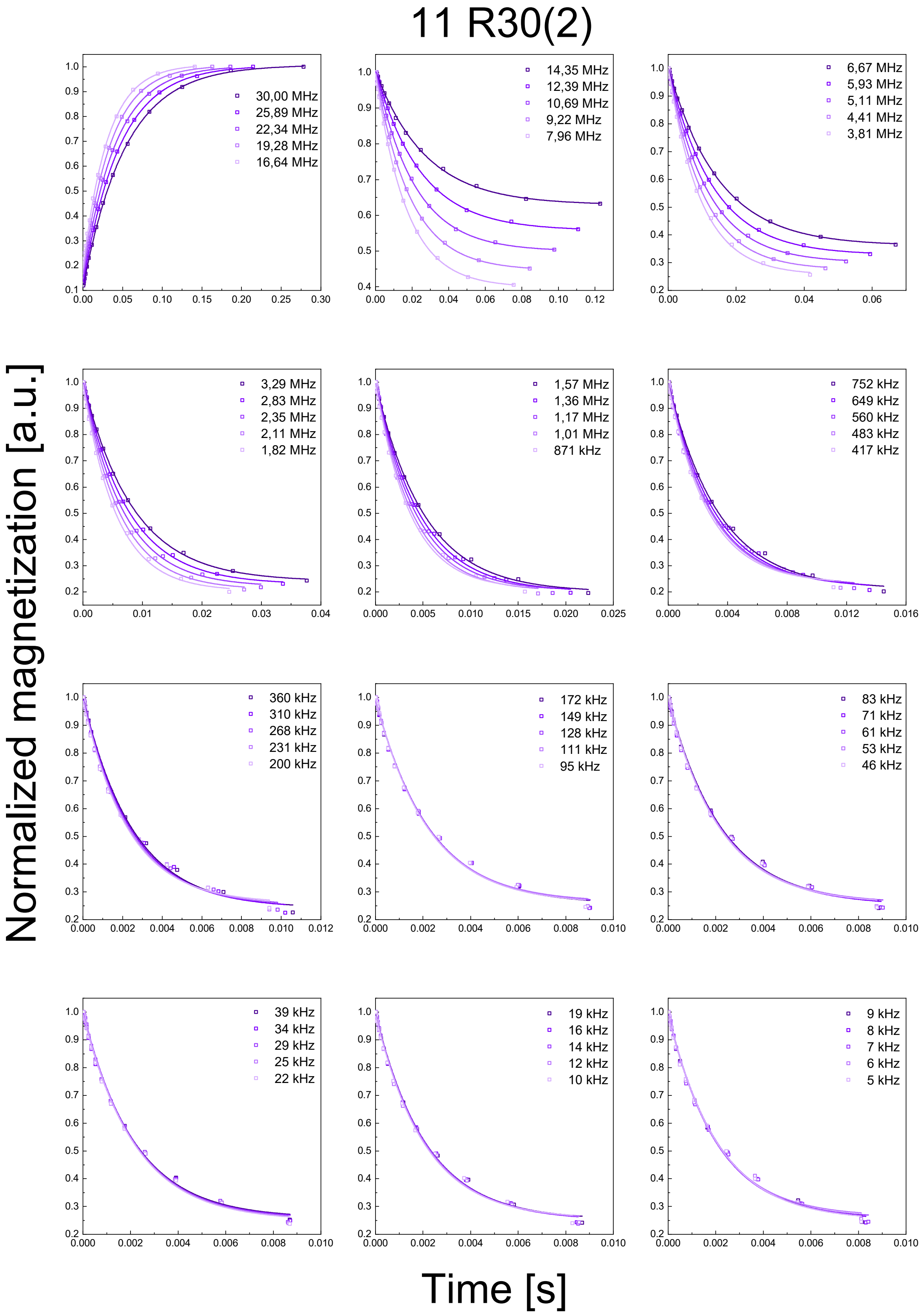
11 R30(2)
Normalized magnetization [a.u.]
Time [s]
30,00 MHz
25,89 MHz
22,34 MHz
19,28 MHz
16,64 MHz
14,35 MHz
12,39 MHz
10,69 MHz
9,22 MHz
7,96 MHz
6,67 MHz
5,93 MHz
5,11 MHz
4,41 MHz
3,81 MHz
3,29 MHz
2,83 MHz
2,35 MHz
2,11 MHz
1,82 MHz
1,57 MHz
1,36 MHz
1,17 MHz
1,01 MHz
871 kHz
752 kHz
649 kHz
560 kHz
483 kHz
417 kHz
360 kHz
310 kHz
268 kHz
231 kHz
200 kHz
172 kHz
149 kHz
128 kHz
111 kHz
95 kHz
83 kHz
71 kHz
61 kHz
53 kHz
46 kHz
39 kHz
34 kHz
29 kHz
25 kHz
22 kHz
19 kHz
16 kHz
14 kHz
12 kHz
10 kHz
9 kHz
8 kHz
7 kHz
6 kHz
5 kHz
